# Wearable Sensor Data Classification for Human Activity Recognition Based on an Iterative Learning Framework †

**DOI:** 10.3390/s17061287

**Published:** 2017-06-07

**Authors:** Juan Carlos Davila, Ana-Maria Cretu, Marek Zaremba

**Affiliations:** Department of Computer Science and Engineering, Université du Québec en Outaouais, Gatineau, QC J8Y 3G5, Canada; ana-maria.cretu@uqo.ca (A.-M.C.); marek.zaremba@uqo.ca (M.Z.)

**Keywords:** large wearable sensor dataset, human locomotion, inertial measurement units, 3-axial acceleration sensors, finite impulse response, wavelet filters, iterative classifier, SVM, multi-class classification

## Abstract

The design of multiple human activity recognition applications in areas such as healthcare, sports and safety relies on wearable sensor technologies. However, when making decisions based on the data acquired by such sensors in practical situations, several factors related to sensor data alignment, data losses, and noise, among other experimental constraints, deteriorate data quality and model accuracy. To tackle these issues, this paper presents a data-driven iterative learning framework to classify human locomotion activities such as walk, stand, lie, and sit, extracted from the Opportunity dataset. Data acquired by twelve 3-axial acceleration sensors and seven inertial measurement units are initially de-noised using a two-stage consecutive filtering approach combining a band-pass Finite Impulse Response (FIR) and a wavelet filter. A series of statistical parameters are extracted from the kinematical features, including the principal components and singular value decomposition of roll, pitch, yaw and the norm of the axial components. The novel interactive learning procedure is then applied in order to minimize the number of samples required to classify human locomotion activities. Only those samples that are most distant from the centroids of data clusters, according to a measure presented in the paper, are selected as candidates for the training dataset. The newly built dataset is then used to train an SVM multi-class classifier. The latter will produce the lowest prediction error. The proposed learning framework ensures a high level of robustness to variations in the quality of input data, while only using a much lower number of training samples and therefore a much shorter training time, which is an important consideration given the large size of the dataset.

## 1. Introduction

Wearable sensor technologies are gaining interest in different research communities due to the use of significantly miniaturized electronic components, with low power consumption, which makes them ideal for applications in human activity recognition for both indoor and outdoor environments [1]. These applications allow users to achieve a natural execution of any physical activity, while providing good results in multiple practical applications, such as health rehabilitation, respiratory and muscular activity assessment, sports and safety applications [2]. However, in practical situations, the collected data are affected by several factors related to sensor data alignment, data losses, and noise among other experimental constrains, deteriorating data quality and model accuracy [3]. Also, the non-ergodicity of the acquisition process, especially when processing signals from acceleration sensors, will result in poor learning performance [4] in applications involving multi-class classification [5]. The problem becomes even more complex if the multi-class classification process is applied on high dimensionality data vectors. Considering these restrictions prevalent in multimodal sensor data fusion [4], which is the case of the work reported in this paper, feature extraction becomes a critical component for finding multi-variable correlations that allow the classifier to improve the model precision while producing a low misclassification rate. 

In this paper, we present a novel method for classifying human locomotion activities, such as walk, stand, lie and sit, by implementing a data-driven architecture based on an iterative learning framework. The proposed solution optimizes the model performance by choosing the best training dataset for non-linear multi-class classification by using an SVM multi-class classifier, while also reducing the computational load. We aim to show that by appropriately choosing the data samples for the training of this multi-class classifier, we can achieve results close to the current approaches reported in literature, while using only a fraction of the data and improving significantly the computation time. The article is organized as follows: Section 2 discusses relevant work on the topic from the literature. Section 3 formalizes and details our method. Section 4 and Section 5 present experimental results, and Section 6 discusses the conclusions.

## 2. Literature Review and Related Works

The new wearable technology used to recognize human activity, based on a wide offer of wireless sensors such as accelerometers, gyroscopes, barometers and other devices with low power consumption, is extremely attractive to customers in a wide area of applications, ranging from fitness to clinical monitoring. Nevertheless, the interpretation of the data collected by such sensors when characterizing the type of activities being executed by a user still brings serious challenges to developers, related to the activity complexity (i.e., certain activities contain similar gestures), to the extraction of relevant features that allow to differentiate the activities, to the data loss that characterizes any wireless transmitter, and to the complex data preprocessing required to deal with the noise inherent in the collected measurements [6]. 

The complex problem of recognizing human activity has motivated different groups of researchers to benchmark different real-world, multi-mode, non-stationary scenarios with wearable sensing solutions. Machine learning provides an excellent approach to improve model accuracy, based on data structures that might dynamically change, while dealing with complex and large datasets acquired from a particular environment [7]. The three most common learning techniques in machine learning are supervised learning, unsupervised learning and semi-supervised learning. Supervised learning occurs when the learner receives a series of labeled examples as training set and makes predictions on previously unseen examples. The problem with this type of learning is the fact that data needs to be labeled, most of the time manually, in order to guide the learning process. In unsupervised learning, the learner receives a series of unlabeled examples as training set and makes predictions for previously unseen examples. This technique is frequently used in problems of clustering and dimensionality reduction. When the learner receives a limited series of labeled and unlabeled examples as training set and makes a prediction on unseen examples, the process is known as semi-supervised learning. The advantage of the latter is the fact that only a limited number of labeled samples are required, instead of an entire labeled training dataset. Semi-supervised as well as supervised learning are used in classification, regression and ranking problems [8]. 

Learning results can be enhanced by using iterative learning procedures. In a wide range of classification problems, especially in those characterized by multimodality and non-ergodicity. Iterative learning extracts training samples from previous instances and then uses them to improve task performance in the next iteration by updating a learning function with the best result. This process reduces the classification error and generates a prediction rule that leads to an improvement of a learned function. In the literature, we can find different examples of iterative learning applications in problems related to text recognition, control, data de-noising and model accuracy improvement [9,10,11,12,13,14]. 

In the context of wearable sensors applications, some results obtained using machine learning techniques are described by Lara and Labrador [15]. The authors reported on human activity recognition systems based on supervised learning approaches, with overall accuracy between 84% and 97.5%, in applications related to exercise analysis and monitoring of patients with heart disease, diabetes and obesity [15], with data gathered on a daily or weekly basis. The authors also reported applications based on semi-supervised learning techniques with an overall accuracy up to 96.5%. Some of these results were obtained by using a training dataset containing 2.5% of the total amount of data, and employing multi-graph algorithms and support vector machines (SVM) combined with multiple eigenspaces. This approach is close to our approach, since we also make use of eigenvalues (scores) produced by principal component analysis (PCA). Other learning techniques, like decision trees, Bayesian and neural networks, fuzzy logic, Markov models and boosting [16] have also shown significant potential in wearable sensing, especially when dealing with problems like segmentation (determined by the variability and the periodicity produced by human activity) and classification [15,17].

This paper is based on our previous work on the topic of human activity classification from wearable sensor data [1]. The classification task is carried out by an iterative learning procedure, where the selection of training samples from previous iterations is guided by the distribution of sample clusters. We are presenting here a novel approach to the problem by using a two-stage consecutive filtering instead of the single stage used in [1]. The objective of the second stage is to enhance the precision of the acceleration signals (i.e., related to activity frequency and motion intensity), and therefore facilitating the process of feature extraction and selection [18,19]. In [1], an iterative learning process is investigated, where data sets associated with each statistical modality are identified through the process of a consecutive selection of the best candidate samples. The iterative process was initially proposed in [13] to solve a regression problem of finding a chlorophyll-a concentration model in inland waters. This paper addresses a classification problem. The learning method and the above changes lead to improved classification results, as it is demonstrated in the experimental section of the paper. 

## 3. Sensor Data Processing

### 3.1. Acquisition of Sensor Data

We address the problem of classifying human locomotion by defining a learning framework based on an iterative learning multi-class classification supported by a multi-class SVM classifier that incorporates the maximum-margin principle to select the best sample candidates. Our framework is experimentally validated on data extracted from the Opportunity dataset [20]. In particular, we are analyzing data acquired from body–worn sensors, as they were recorded in this dataset. The Opportunity dataset has been previously used as a benchmarking reference for modeling different systems, such as labeling large robot-generate activity data sets [21], sensors relocation due to replacement or slippage [22,23], dynamic sensor selection with power minimization [24], and other application-related initiatives [25]. 

According to the Opportunity project’s technical description [26], the body-worn sensors used are twelve 3-axial acceleration sensors and seven inertial measurement units—IMUs (Xsens model MT9). The location of these units is summarized in Table 1 [20]. The dataset has a total of 58 dimensions including the time stamp. Each device senses the acceleration in the three perpendicular axes, recording the acceleration values at the sampling rate of 30 Hz. Records are labeled according to four primitive classes, namely walk, lie, sit and stand. The signal acquisition protocol is performed under a pre-established scenario with six experimental sessions (or runs), performed independently by each of the four users. The extracted dataset contains a total of 869,387 samples, which are distributed as follows: 234,661 samples for user 1; 225,183 samples for user 2; 216,869 samples foruser 3, and 192,674 samples for user 4. 

**Figure 1:**
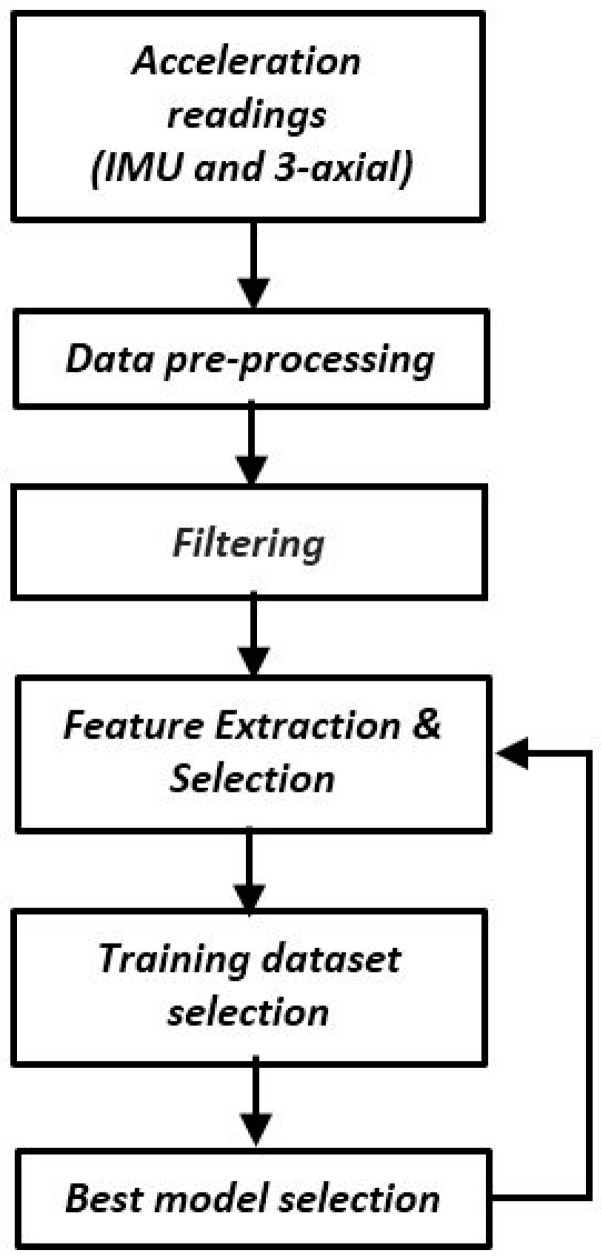
Iterative architecture for multiclass classification.

Our goal is to extract from these data the best training samples that enable the classification of the locomotion activity of the user-dependent models. For this purpose, we are proposing a framework that contains six functional blocks, illustrated in Figure 1, and described in the next sections. 

### 3.2. Data Pre-Processing

The data pre-processing phase consists of two steps. First, we proceed with the exclusion of values affected by data losses and random noise, issues that are very common in wireless acceleration sensors. This represents in the case of our dataset roughly 30% of the sensor readings. To deal with the problem of missing data, we fused—as detailed later in Section 3.3—all readings produced by each sensor, for each user and each experiment, to work exclusively from a data-driven perspective. The aim of the second data-preprocessing step is to filter and de-noise raw data (i.e., sensor readings). In our previous work [1], we used a single filtering stage, based on wavelets. In this work, we are adding an additional finite impulse response (FIR) filter prior to the wavelet filtering stage in order to enhance the precision of the acceleration signals. 

#### 3.2.1. Band-Pass FIR Filtering

In our analysis, high frequency bands are not relevant due to the fact that users are not performing routines with high motion intensity like running, jumping or jogging. Moreover, in general, the acceleration signals present a high level of correlation within a limited-length time window, implying that a FIR filter can be efficiently used in this application [27,28,29]. We use a FIR passband architecture of the order of 40, which is a compromise between the complexity of the signals under observation and the delay introduced by higher orders. Due to the fact that the 3-axial acceleration sensors used sampling frequencies of 32 Hz and 64 Hz, we use cutoff frequencies of 2 Hz and 15 Hz. The frequency of 15 Hz meets the Nyquist theorem ($f_{s}>2\times f_{n}$), where $f_{s}$ is the sampling frequency and $f_{n}$ corresponds to the motion intensity [30]. The frequency of 2 Hz is selected according to criteria presented in [30]. The selected passband provides us with an optimal range of motion intensity due to the fact that the motion recorded in this study does not go beyond 15 Hz, making it acceptable to perform human motion sensing. Once the FIR filtering is processed, we proceed with the second stage—based on wavelets—that is described in the following section. 

#### 3.2.2. Wavelet Filtering

In order to efficiently de-noise raw data, we include a mechanism that guarantees that the resulting classification model is not biased due to the quality of the input data [31]. In general, the acceleration sensors are influenced by several noise sources, such as electrical noise induced by the electronic devices [32], or noise produced by the wireless communication processes, resulting from the propagation phenomenon and causing distortion in the transmitted signal. The noise present in the acceleration sensor measurements has commonly a flat spectrum. It is present in all frequency components, constituting a serious challenge for the use of traditional filtering methods, which by removing sharp features, can introduce distortions in the resulting signal. Decomposition of the noisy signal into wavelets [33] eliminates small coefficients, commonly associated with the noise, by zeroing them, while concentrating the signal in a few large-magnitude wavelet coefficients. Wavelet filtering consists in the decomposition of the signal into wavelet basis functions $\psi_{a,b}\left(t\right)$ given by [34]:

(1) $$\psi_{a,b}\left(t\right)=\frac{1}{\sqrt{a}}\psi \left(\frac{t-b}{a}\right)$$

where a,b∈R are called scale and position parameters respectively. The wavelet basis is defined by the selection of the previous parameters. Their choice is commonly known as critical sampling, hence, $a=2^{-j}\;\mathrm{and}\;b=\left(k\right)2^{-j}$, where k and j are integers, will give a spare basis [35]. The function in Equation (1) can be represented in powers of two; this strategy is called dyadic and can be formulated as:

(2) $$\psi_{m,n}\left(k\right)=2^{\frac{-m}{2}}\psi \left(2^{-m}k-n\right)$$

where m,n∈ℤ. By computing an inner product between any given function f(k) and $\psi_{m,n}\left(k\right)$, we can obtain the wavelet transform as:

(3) $$\mathrm{DWT}\left(m,n\right)=\langle f,\psi_{m,n}\rangle =2^{\frac{-m}{2}}\overset{\infty }{\underset{k=-\infty }{\sum }}f\left(k\right)\psi \left(2^{-m}k-n\right)$$

The advantage of having a function represented in wavelets is the flexibility of the mathematical model, defined in the domain of both frequency and time, in the frequency domain via dilation and in the time domain via translation. This feature is helpful also when removing noise, because the main characteristics of the original signal can be more easily preserved. Wavelet de-noising involves thresholding of a range of wavelet coefficients. Setting wavelet coefficients below a specific value (λ) to zero [34] is called hard-thresholding and it can be represented as:

(4) $$f\left(k\right)=\{\begin{array}{l} k\;\mathrm{if}\;\left|k\right|>\;\lambda \\ 0,\;\mathrm{otherwise} \end{array}$$

In addition, if the wavelet coefficients are below the threshold value, they are shrunk, and when the coefficients are above the threshold value, they are scaled. This process is called soft-thresholding and can be represented as:

(5) $$f\left(k\right)=\max\left(0,1-\frac{\lambda }{\left|k\right|}\right)$$

In the literature, we can find four well-known threshold estimation methods [34], namely the Minmax criterion [35], the Square root log (SQTWOLOG) criterion [35], the Rigrsure criterion [36] and the heursure criterion. In general, the correct selection of the threshold leads to a better noise suppression; a large threshold value will bias the estimator, while a low value will increase the variance. The thresholding approach selected in this work employs the SQTWOLOG criterion, because it guarantees a high signal-to-noise ratio (SNR) with a low mean square error (MSE). The threshold values are calculated by the universal threshold $\sqrt{2\times \ln\left(N\right)}$ where N is the length of the signal, or $\lambda_{i}=\sigma_{j}\sqrt{2\log\left(N_{j}\right)}$, where $N_{j}$ is the length of the noise at $j^{\mathrm{th}}$ scale and $\sigma_{j}$ is the Median Absolute Deviation (MAD) at the $j^{\mathrm{th}}$ scale given by [34]:

(6) $$\sigma_{j}=\frac{{\mathrm{MAD}}_{j}}{0.6745}=\frac{\mathrm{median}\left(\left|\omega \right|\right)}{0.6745}$$

where ω represents the wavelet coefficients at scale j. The value 0.6745 in Equation (6) is obtained as: $\frac{1}{\mathrm{erf}\left(0.5\right)*\sqrt{2}}$, where the Gauss error function (erf) is computed by integrating the normal distribution. This value will scale the MAD to obtain an approximation for sigma (only for a Gaussian distribution).

### 3.3. Feature Extraction and Selection

After filtering the raw data, we proceed with the feature extraction and selection process. The aim is to retrieve a set of data with high correlation, allowing us to extract the best candidates for the training dataset [37]. This process focuses on the extraction of kinematics features, such as roll, pitch, yaw (RPY), and the norm of the axial components produced by each of the body–worn sensors. Our first feature set is based on the signal magnitude vector (SMV). At each time instance j, the acceleration sensor k produces a 3-axial vector, consisting of acceleration values along a system of orthogonal axes $a_{j,k}=\left({\mathrm{acc}}_{x},{\;\mathrm{acc}}_{y},{\;\mathrm{acc}}_{z}\right)\in {ℛ}^{3}$. For each sensor, we can retrieve the single magnitude vector $\left|a_{j,k}\right|$. The second feature set is related to roll, pitch and yaw (RPY) angles, calculated as follows:

(7) $${\mathrm{roll}}_{j,k}=\mathrm{atan}\left(\frac{{\mathrm{acc}}_{x}}{\sqrt{{\mathrm{acc}}_{y}+{\mathrm{acc}}_{z}}}\right);\;{\mathrm{pitch}}_{j,k}=\mathrm{atan}\left(\frac{{\mathrm{acc}}_{y}}{\sqrt{{\mathrm{acc}}_{x}+{\mathrm{acc}}_{z}}}\right);{\;\mathrm{yaw}}_{j,k}=\mathrm{atan}\left(\frac{{\mathrm{acc}}_{z}}{\sqrt{{\mathrm{acc}}_{x}+{\mathrm{acc}}_{y}}}\right)$$

Finally, we build a matrix with all axial components produced by all sensors under observation:

(8) $${\mathrm{acc}}_{x,y,z,k}=\left\{\left[{\mathrm{acc}}_{x,k}\right],\left[{\mathrm{acc}}_{y,k}\right],\left[{\mathrm{acc}}_{z,k}\right]\right\}$$

This matrix has $n\times a_{j,k}\times k$ components, where n is the number of samples in each experiment for k sensors in $a_{j,k}$ dimensions. To deal with the absence of some values, we use principal component analysis (PCA) and singular value decomposition (SVD). PCA provides a mechanism to reduce dimensionality, while SVD provides a convenient way to extract the most meaningful data. Combining these techniques, we find data dependency while removing redundancy. PCA [38] and SVD [39] ensure the preservation of the nature of the resulting data structures in each feature category. When applying PCA, each feature is compressed in two principal components as presented in Section 4.1. Similarly, when SVD is applied, each feature is reduced to two SVD dimensions, as shown in Equation (9). The new target function $f_{j,k}()$ is represented as follows:

(9) $$f_{j,k}=f\left(\mathrm{pca}\;\left(\mathrm{RPY}\right),\mathrm{pca}\left(\mathrm{SMV}\right),\mathrm{pca}({\mathrm{acc}}_{x,y,z,k}),\mathrm{svd}\left(\mathrm{RPY}\right),\mathrm{svd}\left(\mathrm{SMV}\right),\mathrm{svd}\left({\mathrm{acc}}_{x,y,z,k}\right)\;\right)$$

where j corresponds to each observation produced by sensor k. We are therefore reducing our analysis to a function with three attributes ($\mathrm{RPY},\mathrm{SMV},{\mathrm{acc}}_{x,y,z,k}$) and two mathematical methods, PCA and SVD.

## 4. Iterative Learning Architecture

Our learning framework aims to classify human activities using a single multi-class SVM classifier [40] (LibSVM version 3.20 for Matlab). To achieve this, we must deal with two data constrains: (1) the large size of the experimental dataset, containing in many cases overlapping class members and high data density; and (2) the non-ergodicity of the recorded signals. In order to improve the classification accuracy, while keeping the required processing time at the minimum, features $\;((f_{1},f_{2}),\ldots ,\left(f_{j},f_{k}\right))$ produced by Equation (9) are grouped pairwise to cover all the possible combinations. The candidates for the training dataset are then determined by measuring the Euclidean distance between each class member and the centroids of each distribution of ($f_{j},f_{k}$). If the resulting distance is larger than the mean plus the standard deviation of all resulting Euclidean distances, then the class member is considered a candidate for the training set. This process leads to the creation of support vectors, which generate the optimal separation plans to classify the remaining data with only a fraction of the total data presented for each user experiment. The goal is to build a robust classification model, which will not be affected by the quality of the input data [41]. 

### 4.1. Training Data Selection

**Figure 2:**
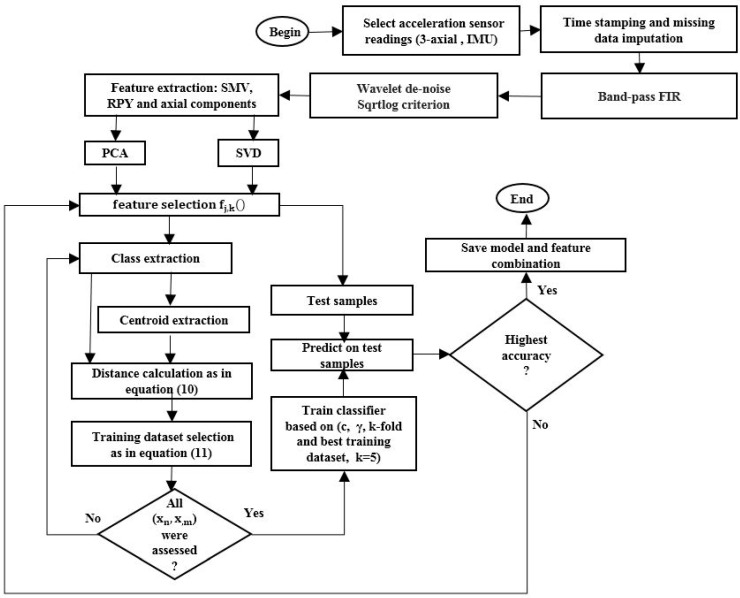
Iterative architecture for multiclass classification.

**Figure 3:**
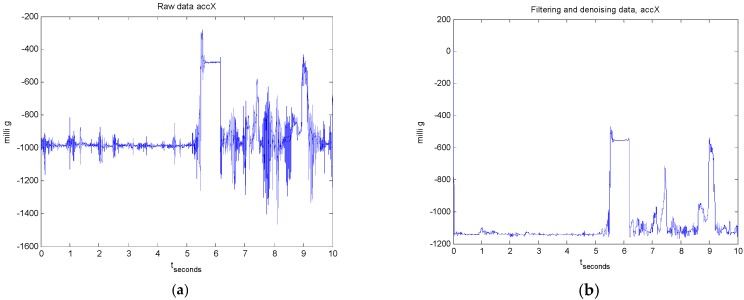
Measurements recorded for user 1 and experiment 1 for a 3-axial acceleration sensor located on the up right knee: (**a**) raw data; and (**b**) after applying 2-stage filtering.

**Figure 4:**
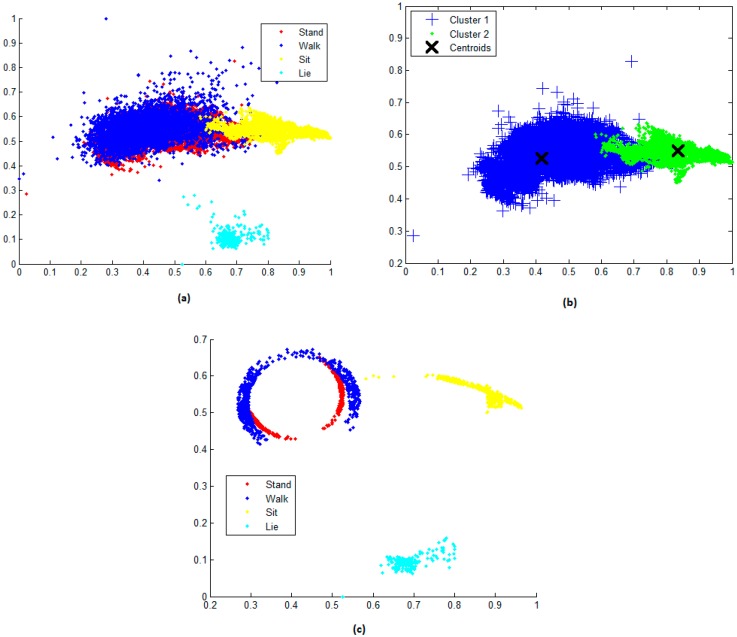
(**a**) PCA is applied to ${\mathrm{acc}}_{x,y,z,k}$ (data distribution corresponds to the first and second principal components); (**b**) Classes are extracted in pairs ($x_{n},x_{,m})$, centroids are extracted and Euclidean distances are calculated according to step 6; and (**c**) Training candidates are produced by the selection algorithm.

The following procedure, illustrated in detail in Figure 2, summarizes the process for the extraction of the training dataset (for any user and any experiment):

1 Select sensor readings recorded (in this case, from the Opportunity dataset [20]), perform time stamping and missing-data imputation (Figure 2).
2 Select band-pass FIR filter (2–15 Hz) and perform wavelet de-noising using SQTWOLOG criterion (Figure 3).
3 Extract kinematics features: signal magnitude vector, roll, pitch, yaw (RPY), and the norm of the axial components produced by each of the body–worn sensors, in order to create the target function $f_{j,k}()$ as indicated in Equation (9). This step will produce twelve features.
4 Build a subset of features ($f_{j},f_{k}$), where j=(1,…,11) and k=(2,…,12) from target function $f_{j,k}()$ and extract classes presented in subset ($f_{j},f_{k}$) (Figure 4a).
5 Select a pair of classes ($x_{n},x_{,m})$, from subset ($f_{j},f_{k}$) where n=(1,…,l−1) and m=(2,…,l) and l is the number of labels in the dataset (in our case four classes corresponding to each locomotion activity), and extract centroids produced by members of each class.
6 Extract the Euclidean distance between each class member in ($x_{n}$) and the centroid of the class ($x_{m}$). Store the results in a vector of distances $R_{n,m}\left(j\right)$:
  (10) $$R_{n,m}\left(j\right)=\left|\left(x_{n,m}\left(j\right)\right)-{\mathrm{Centroid}}_{n,m}\right|\;$$
  where n and m are the classes of ($f_{j},f_{k}$), j is a class member and ${\mathrm{Centroid}}_{n,m}$ is the opposite centroid, with respect to the discriminating hyperplane, of the class member under evaluation (Figure 4b).
7 If the resulting Euclidean distance vector $R_{n,m}\left(j\right)$ satisfies condition (11), then the class member is a candidate for the training dataset.
  (11) $$R_{n,m}\left(j\right)\geq \bar{R_{n,m}}+\sigma (R_{n,m})$$
  where $\bar{R_{n,m}}$ and $\sigma \left(R_{n,m}\right)$ are the mean and standard deviation of the Euclidean distance vector $R_{n,m}\left(j\right)$. The candidate is stored in a vector of candidates (VoC), VoC($x_{n,m}\left(j\right)$) (Figure 4c).
8 Repeat steps 9 to 12 until n=l−1 and m=l.
9 Repeat steps 7 to 13 until j=11 and k=12.

Figure 4a shows the data distribution when PCA is applied to features generated by axial components from the sensor measurements, for example, for the first two PCA components $f_{1,2}=f\left(\mathrm{pca}\left({\mathrm{acc}}_{x,y,z,k}\right)\right)$. Both components are called scores. The advantage of PCA is that the resulting score does not change the order of the original rows (observations), helping us to preserve the previously assigned labels. In this figure, we also observe a clear separation between the sit (shown in yellow) and the lie (shown in cyan) instances, while the stand (shown in red) and the walk (shown in blue) classes overlap. Permutation of the members from $f_{j,k}$ helps us to find different data distributions from the original data structure. This provides some distributions with linearly separable data, which decreases the misclassification error rate produced by the multi-class classifier. 

Figure 4b represents the extraction of two classes ($x_{n},x_{,m})$ from $f_{j,k}$ and their respective clusters. Our goal is to extract the samples producing the largest Euclidean distances as measured between each sample and its opposite centroid. This operation is processed by pairing the classes (stand = 1, walk = 2, sit = 3 and lie = 4). 

Figure 4c shows the resulting VoC($x_{n,m}\left(j\right)$) composed by samples that satisfy Equation (11), that is: VoC($x_{n,m}\left(j\right)$) = $\left[\left({\mathrm{Class}}_{1},{\mathrm{Class}}_{1}\right),\left({\mathrm{Class}}_{1},{\mathrm{Class}}_{2}\right),\ldots ,\left({\mathrm{Class}}_{n-1},{\mathrm{Class}}_{m}\right)\right]$, where n, m = 4. This mechanism provides an effective way to deal with non-separable data (data overlapping). Because the SVM classification depends only on the training samples near the decision boundary, the optimal separation margin will be determined by the separation of the training samples controlled by the cost parameter C [13]. The improvement can be observed by comparing the separation on Figure 4c with Figure 4a, where we notice a strong overlapping of data samples, in particular for the stand, walk and sit classes.

### 4.2. Model Selection

Once the best training dataset VoC($x_{n,m}\left(j\right)$) is identified, we proceed with the selection of the best classification model using a multi-class SVM classifier with an RBF kernel [1]. The training and testing samples are normalized in the range of 0 to 1 [40]. The kernel selection is done based on an experimental performance evaluation with different kernels, e.g., linear, cubic polynomial and sigmoid. The evaluation presented in [13] and confirmed by initial tests on the Opportunity dataset indicate that RBF kernels consistently produce models with the lowest or close to the lowest misclassification error rates. In this paper, we deal with a multi-class classification problem. The selection of the one-versus-all (OVA) classification method reduced our problem to a multiple binary classification problem. Designing the SVM classifier requires to find the best combination of the cost and gamma (C, γ) parameters. These parameters are extracted from a k-fold cross validation process with k = 5 (using four subsets for training and one subset for testing). This process allows us to find a tradeoff between bias and variance by adjusting C and γ. In order to find the best C and γ we use a grid search, where $C=(2^{-5},\ldots ,2^{7}$) and$\;\gamma =(2^{-5},\ldots ,2^{7}$). In practical terms, the best combination, in the sense of a high variance and a low bias, is that of large C with small γ.

The resulting model is then used to predict the labels on the testing dataset. Once the classification rate is determined, the algorithm stores the accuracy values, features ($f_{j},f_{k}$), C, γ and the size of the VoC($x_{n,m}\left(j\right)$), and repeats the process until all combinations of $\left(f_{j},f_{k}\right)$ are exhausted.

## 5. Experimental Results

**Figure 5:**
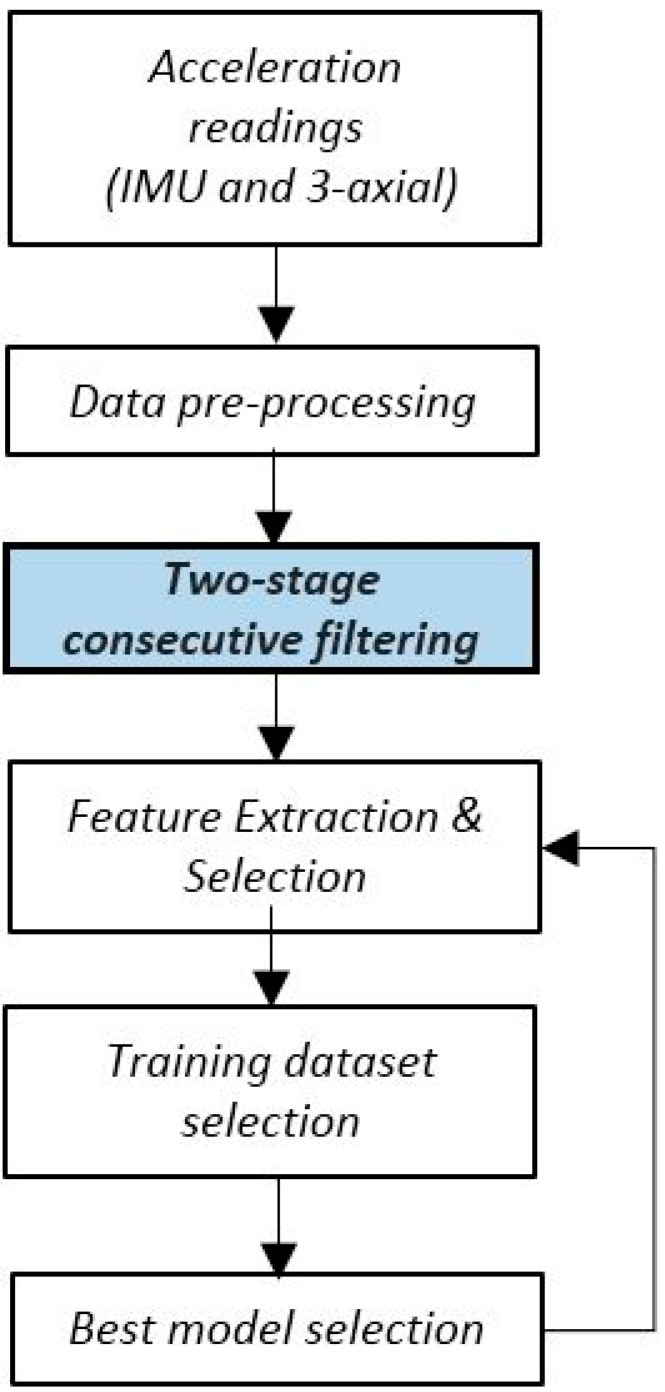
Scenarios with and without the new filtering stage.

The proposed solution, based on iterative learning, is tested in two scenarios, one focusing on a single-stage filtering, such as previously presented in Section 2.1.1 of our conference paper [1], and the other one on a two-stage consecutive filtering, as detailed in Section 3.2.1 and Section 3.2.2 at the present article. The difference between the two filtering cases is shown in Figure 5.

### 5.1. Results Obtained Using Single-Stage Wavelet Filtering

The proposed process was evaluated initially using a single wavelet filtering stage in three experiments: two considering the measurements of a sole sensor and one combining the use of various sensors. Two measures were used to validate the results, namely the prediction accuracy (Acc) and the size (as percentage of the total dataset) of the training dataset that was used for classification (TS):

(12) $$\mathrm{Acc}=\frac{\mathrm{Labels}\;\mathrm{correctly}\;\mathrm{predicted}}{\left(\mathrm{size}\;\mathrm{of}\;\mathrm{user’s}\;\mathrm{dataset}\right)}\times 100\%;\;\mathrm{TS}=\frac{\mathrm{size}\left(R_{n,m}\right)}{\left(\mathrm{size}\;\mathrm{of}\;\mathrm{user’s}\;\mathrm{dataset}\right)}\times 100\%$$

It is important to note that the values of Acc and TS depend on the size of the user dataset and the resulting value of $R_{n,m}\left(j\right)$ in Equation (11). These values are changing with the number of measurements in each user experiment. Table 2 presents the results when using only data obtained from the IMU sensors, Table 3 shows the values for Acc and TS when using data obtained from 3-axial acceleration sensors, and Table 4 when using data obtained when fusing measurements from the 3-axial acceleration sensors and IMU devices in three experiments. The results obtained by our iterative learning framework are compared with the case in which 80% of total of data are used of each user experiment, which is a common practice when a k-fold cross-validation process is performed, with k = 5. In this case, the samples are randomly selected from the input domain. 

**Figure 6:**
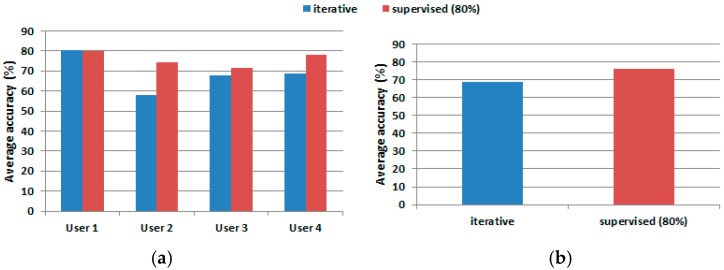
Accuracy comparison: (**a**) accuracy generated by SVM multi-class classifier on each user; and (**b**) average accuracy for iterative versus supervised methods.

**Figure 7:**
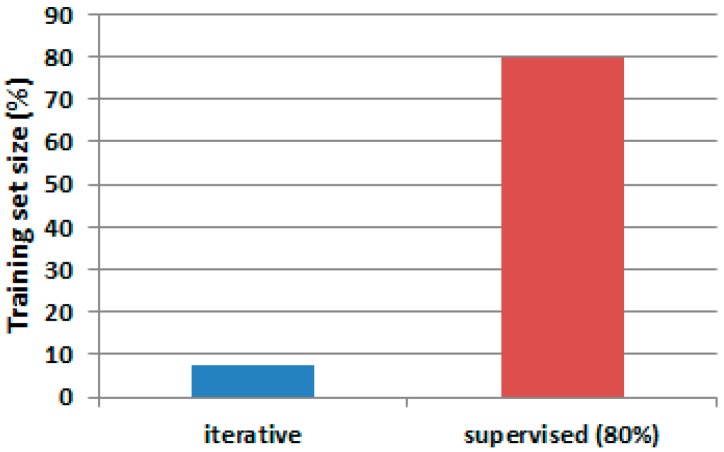
Training size comparison.

These results are compared graphically in Figure 6 that shows the average accuracy when using two training dataset selection strategies: iterative with a limited number of training samples (in blue), and supervised one with a large number of training samples (in red). One can observe that using on average 7.33% of the dataset for training (Figure 7), the performance achieved is only 7.28% under the performance obtained when the classifier processes a high number of training samples. 

The use of a smaller training set leads as well to an important decrease in the computation time. The average processing time per user is roughly 35 min when using the training with 80% of the dataset (Matlab running on a single processor Intel 7 CPU with 6 Gb RAM memory). The use of the iterative process leads to a reduction in the average time for processing an experiment to about 5 min, which is less than 15% of the time required by the fully supervised process. 

### 5.2. Results Obtained Using Two-Stage Consecutive Filtering

In this section, we present the experimental results when the bandpass FIR filter and subsequently wavelet de-noising are applied on the data collected from IMU sensors, 3-axial acceleration sensors and when fusing measurements from the IMU and 3-axial acceleration sensors (Table 5, Table 6 and Table 7). These values are co with results obtained in Section 5.1. As detailed in Section 3.1 and Section 3.2, it is expected that performance will increase as a result of this two-stage consecutive filtering. 

In general, we noticed a performance improvement when the framework uses a two-stage consecutive filtering. Deployment of the extra filtering stage generated an increase in the average accuracy. For example, for User 2, an average accuracy of 61.40% is obtained with wavelet filtering (Table 5). An average accuracy of 67.71% is obtained with two-stage consecutive filtering, which corresponds to an improvement of 6.30%. Similarly, in Table 6, an average improvement of 12.72% can be noticed. Finally, in Table 7, for the same user we obtained an improvement of 12.24%. 

Results obtained by using a training data set of 80% of total data are summarized in Table 8, Table 9 and Table 10. Better results are obtained when classification is performed on fused data coming from IMU and 3-axial acceleration sensors.

**Figure 8:**
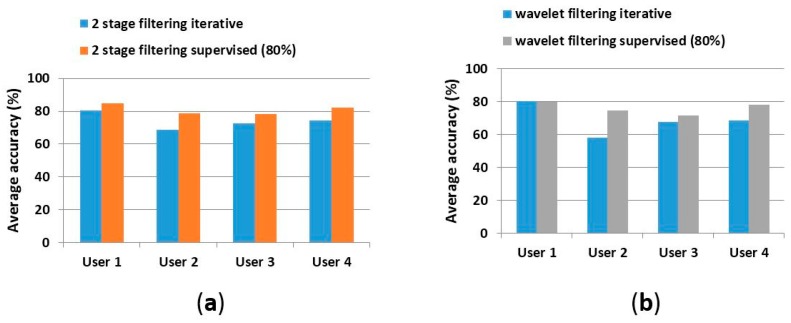
Average accuracy comparison between single-stage and two-stage filtering. (**a**) average accuracy when using two-stage filtering and the iterative methodology (in blue) and when using the supervised method (in orange); and (**b**) average accuracy when using the single-stage (wavelet filtering) solution and iterative methodology (in blue) and when using the supervised method (in grey).

Figure 8 presents an accuracy comparison between the single-stage approach [1] and the two-stage filtering process. 

**Figure 9:**
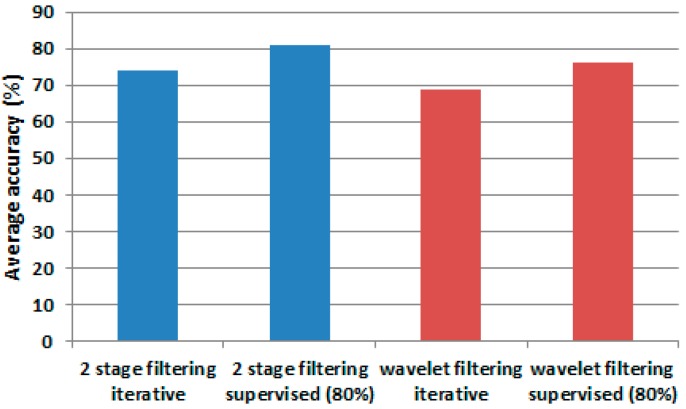
Average accuracy. Bars in blue represent average accuracy when two-stage filtering is used. Bars in red represent the results for single-stage wavelet filtering.

The approach with two-stage filtering, as compared with the wavelet filtering only, generated an accuracy improvement in those experiments where only a fraction of samples was used for training. Overall, the second filtering produced an average accuracy of 74.08% versus 68.76% produced by the single filtering approach [1], an equivalent of 5.32% of improvement. The model accuracy for user 2 was improved by 6.11% for readings obtained from 3-axial acceleration sensors and by 3.88% when IMU and 3-axial acceleration sensors were fused. The performance was improved by 5.03% for the case of the training size of 80% of the total amount of the input data (Figure 9). 

### 5.3. F-Measure Results when Using Two-Stage Consecutive Filtering

In previous experiments, we presented the results based on how effective the algorithm was in predicting the true values of a label. In this section, we quantify the classification results using the $F_{1}\;\mathrm{score}$ [42], which takes into account recall and precision metrics. Precision is defined as the ratio of true positive (TP) divided by the sum of the TP and false positives (FP), while recall will be the radio between TP divided by the sum of TP and false negatives (FN). In a general case, the $F_{1}\;\mathrm{score}$ is defined as [42]:

(13) $$F_{\beta }=\frac{\mathrm{Precision}\times \mathrm{recall}\times \left(1+\beta^{2}\right)}{\mathrm{Precision}+\mathrm{recall}\times \beta^{2}}$$

where β is the parameter that controls the importance given to the precision and recall. In our case, we give equal importance to both metrics (β=1), therefore, F-measure is defined as:

(14) $$F_{1}=\frac{2\times \mathrm{precision}\times \mathrm{recall}}{\mathrm{precision}+\mathrm{recall}}$$

By applying the result to each class, we have [2]:

(15) $$F_{1}=\underset{i}{\sum }2\times \frac{{\mathrm{precision}}_{i}\times {\mathrm{recall}}_{i}}{{\mathrm{precision}}_{i}+{\mathrm{recall}}_{i}}\times w_{i}$$

where i is the class index, $w_{i}=\frac{n_{i}}{N}$, N is the total number of samples, and $n_{i}$—the number of samples of the ith class. The results are presented in Table 11.

**Figure 10:**
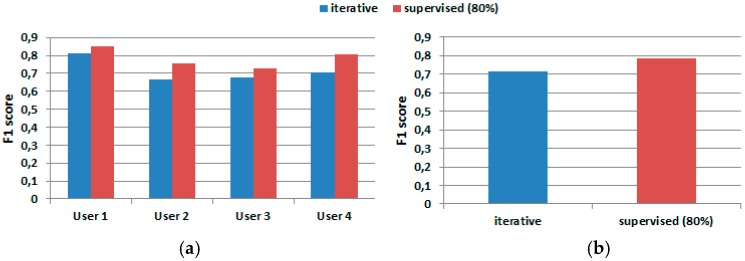
$F_{1}\;\mathrm{score}$ comparison for IMU and 3-axial acceleration sensors fused data. (**a**) Results for each user; and (**b**) average $F_{1}\;\mathrm{score}$.

Figure 10 presents the $F_{1}\;\mathrm{score}$ for both learning schemes. One can notice a total average difference of 0.075 between the two methods, as compared to 0.0532 in Table 11. 

**Figure 11:**
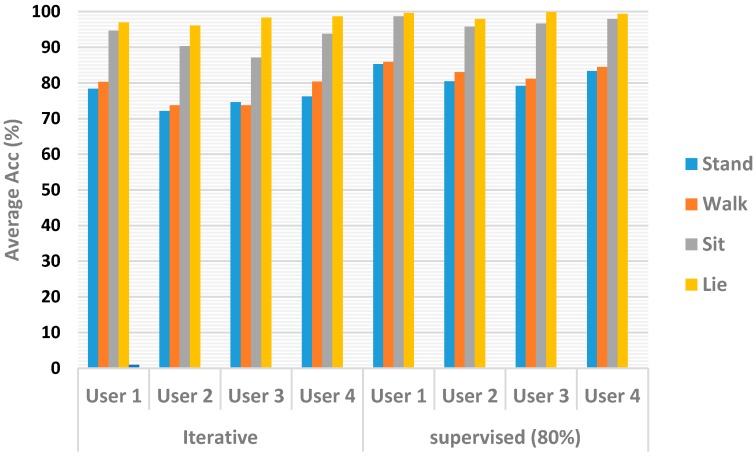
Classification model accuracy comparison between iterative and supervised methods.

Finally, the performance of our algorithm was evaluated for each class. Figure 11 shows the average accuracy obtained for each user. One can notice a marked separation between the sit and lie activities versus walk and stand. The difficulty in distinguishing walk from stand stems from the overlapping of data. The iterative method produced an average accuracy of 75.4% for the walk movement, compared with 82.1% obtained by the supervised method. Similarly, for the stand activity, the iterative method produced an average accuracy of 77.06%, which makes a difference of 6.57% with respect of the value obtained by the supervised method (83.63%). However, the classification difference is reduced for the lie activity—an average accuracy of 97.57% for the iterative method and 99.18% for the supervised one. When classifying the sit activity, the iterative process produced an average accuracy of 91.46% while the supervised method produced 97.27%. 

## 6. Conclusions

In this paper, we proposed a novel iterative learning process to reduce the number of samples and subsequently the processing time for the classification of human activities from wearable sensor measurements. The challenges related to the large percentage of missing data and the noise affecting the measurements were successfully dealt when applying data fusion with a robust two stage filtering mechanism combined with an iterative learning process. Our iterative learning framework produced an average accuracy of 74.08% while using only 6.94% of the samples in the input domain for training. This result compares to the average accuracy of 81.07% obtained by the supervised method when using 80% of samples for training and the 20% remaining samples for testing. The need for significantly less data entails much shorter computation times. The additional FIR filtering stage and the wavelet filtering resulted in a substantial average improvement for some user data models (e.g., user 2) with up to 13.74%, due to the elimination of spurious values produced by noise and other environmental phenomena. The inclusion of a mechanism for the selection of the training dataset allows us to work with only a fraction of the total dataset (average of 6.44%) used in the SVM multi-class training process. The minimization of the number of samples is an important contribution that allows the user to deal efficiently with an ever-growing number of large data sets.

## Figures and Tables

**Table 1:** Placement of sensors (as specified in the Opportunity activity recognition dataset [20]).

Placement	Sensor	
	IMU	3-Axial
Left Foot	1	
Right Foot	1	
Up Right Knee		1
Low Right Knee		1
Hip		1
Back	1	1
Right Forearm	1	2
Left Forearm	1	2
Right Arm	1	
Left Arm	1	
Right Hand		1
Left Hand		1
Right Wrist		1

**Table 2:** Classification performance obtained for IMU sensors.

	Experiments					
	Experiment 1 (Acc%/TS%)	Experiment 2 (Acc%/TS%)	Experiment 3 (Acc%/TS%)	Experiment 1 (Acc%/80%)	Experiment 2 (Acc%/80%)	Experiment 3 (Acc%/80%)
User 1	80/4.47	75.36/1.19	81/3.31	83.92	74.76	80.55
User 2	71.56/4.97	47.43/11.96	65.23/10.18	77.53	77.17	78.31
User 3	70,64/5.70	57/7.70	73.28/0.16	71.46	69.43	75.19
User 4	66.19/2.8	61.27/2.70	78 /1.86	77.2	74.46	79.88

**Table 3:** Classification performance obtained from 3-axial acceleration sensors.

	Experiments					
	Experiment 1 (Acc%/TS%)	Experiment 2 (Acc%/TS%)	Experiment 3 (Acc%/TS%)	Experiment 1 (Acc%/80%)	Experiment 2 (Acc%/80%)	Experiment 3 (Acc%/80%)
User 1	82.82/3.03	79.23/11.38	83.71/9.11	83.12	79.12	80.56
User 2	52.42/2.96	50.86/12	57.84/1.89	69.9	75	73.56
User 3	69/13.16	67.86/0.60	76.62/3.37	72.09	65.21	77.51
User 4	66/1.63	64/10.4	77.53/3.45	71.59	76.15	87.55

**Table 4:** Classification performance obtained from IMU and 3-axial acceleration sensors.

	Experiments					
	Experiment 1 (Acc%/TS%)	Experiment 2 (Acc%/TS%)	Experiment 3 (Acc%/TS%)	Experiment 1 (Acc%/80%)	Experiment 2 (Acc%/80%)	Experiment 3 (Acc%/80%)
User 1	80.62/7.15	77.21/8.3	84.77/8.17	81.11	75.92	80.85
User 2	65.85/8.78	45.16/12.49	66.25/0.90	71.54	76.68	74.56
User 3	58.49/13.93	67.62/1.42	70.35/2.97	72.30	65.18	77.08
User 4	66.48/0.70	66.64/11.41	71.54/4.14	73.43	75.80	87.38

**Table 5:** Classification performance obtained from IMU sensors: filtering comparison.

	Two-Stage Consecutive Filtering			Wavelet Filtering		
	Experiment 1 (Acc%/TS%)	Experiment 2 (Acc%/TS%)	Experiment 3 (Acc%/TS%)	Experiment 1 (Acc%/TS%)	Experiment 2 (Acc%/TS%)	Experiment 3 (Acc%/TS%)
User 1	80.23/5.5	79.5/6.05	80/5.9	80/4.47	75.36/1.19	81/3.31
User 2	76/8.19	50.23/13.8	76.91/6.18	71.56/4.97	47.43/11.96	65.23/10.18
User 3	73.55/5.8	68.22/5.68	76/6.01	70,64/5.70	57/7.70	73.28/0.16
User 4	75.62/4.23	67.71/5.11	72.85/13.79	66.19/2.8	61.27/2.70	78 /1.86

**Table 6:** Classification performance obtained from obtained from 3-axial acceleration sensors: filtering comparison.

	Two-Stage Consecutive Filtering			Wavelet Filtering		
	Experiment 1 (Acc%/TS%)	Experiment 2 (Acc%/TS%)	Experiment 3 (Acc%/TS%)	Experiment 1 (Acc%/TS%)	Experiment 2 (Acc%/TS%)	Experiment 3 (Acc%/TS%)
User 1	81.93/6.05	73.5/6.05	81.48/5.9	82.82/3.03	79.23/11.38	83.71/9.11
User 2	63.25/5	66.53/14	72.50/12.72	52.42/2.96	50.86/12	57.84/1.89
User 3	68.38/7.4	71.60/5.29	78.44/5.46	69/13.16	67.86/0.60	76.62/3.37
User 4	73.63/6.67	72.07/6.33	79.80/6.03	66/1.63	64/10.4	77.53/3.45

**Table 7:** Classification performance obtained from IMU and 3-axial acceleration sensors: filtering comparison.

	Two-Stage Consecutive Filtering			Wavelet Filtering		
	Experiment 1 (Acc%/TS%)	Experiment 2 (Acc%/TS%)	Experiment 3 (Acc%/TS%)	Experiment 1 (Acc%/TS%)	Experiment 2 (Acc%/TS%)	Experiment 3 (Acc%/TS%)
User 1	87.26/6.28	78/5.47	82.30/6.39	80.62/7.15	77.21/8.3	84.77/8.17
User 2	67.5/7.2	71.50/6.40	75/7.46	65.85/8.78	45.16/12.49	66.25/0.90
User 3	74.45/5.12	70.82/5.40	71.67/5.69	58.49 /13.93	67.62/1.42	70.35/2.97
User 4	74.20/7.18	73/7.74	81.41/7	66.48/0.70	66.64/11.41	71.54/4.14

**Table 8:** Classification performance obtained from IMU sensors: filtering comparison.

	Two-Stage Consecutive Filtering			Wavelet Filtering		
	Experiment 1 (Acc%/80%)	Experiment 2 (Acc%/80%)	Experiment 3 (Acc%/80%)	Experiment 1 (Acc%/80%)	Experiment 2 (Acc%/80%)	Experiment 3 (Acc%/80%)
User 1	89.88	77.33	89.33	83.92	74.76	80.55
User 2	84.83	82.36	84.17	77.53	77.17	78.31
User 3	81.79	83.55	85.76	71.46	69.43	75.19
User 4	86.19	84	89.41	77.2	74.46	79.88

**Table 9:** Classification performance obtained from 3-axial acceleration sensors: filtering comparison.

	Two-Stage Consecutive Filtering			Wavelet Filtering		
	Experiment 1 (Acc%/80%)	Experiment 2 (Acc%/80%)	Experiment 3 (Acc%/80%)	Experiment 1 (Acc%/80%)	Experiment 2 (Acc%/80%)	Experiment 3 (Acc%/80%)
User 1	83.42	79.85	82.36	83.12	79.12	80.56
User 2	69.68	76.05	77.90	69.9	75	73.56
User 3	72.30	69.41	82.33	72.09	65.21	77.51
User 4	76.90	74.36	82.21	71.59	76.15	87.55

**Table 10:** Classification performance obtained from IMU and 3-axial acceleration sensors: filtering comparison.

	Two-Stage Consecutive Filtering			Wavelet Filtering		
	Experiment 1 (Acc%/80%)	Experiment 2 (Acc%/80%)	Experiment 3 (Acc%/80%)	Experiment 1 (Acc%/80%)	Experiment 2 (Acc%/80%)	Experiment 3 (Acc%/80%)
User 1	91.43	79.64	88.32	81.11	75.92	80.85
User 2	74.51	79.93	79.98	71.54	76.68	74.56
User 3	78.97	68.91	82.92	72.30	65.18	77.08
User 4	82.97	78.66	86.85	73.43	75.80	87.38

**Table 11:** $F_{1}\;\mathrm{score}$ for data fused from IMU and 3-axial acceleration sensors.

	Experiments					
	Experiment 1 ($F_{1}$/TS%)	Experiment 2 ($F_{1}$/TS%)	Experiment 3 ($F_{1}$/TS%)	Experiment 1 ($F_{1}$/80%)	Experiment 2 ($F_{1}$/80%)	Experiment 3 ($F_{1}$/80%)
User 1	0.8506/6.28	0.7669/5.47	0.79/6.39	0.9103	0.7701	0.8786
User 2	0.62/7.22	0.6809/6.40	0.695/7.46	0.7324	0.7821	0.7545
User 3	0.7283/5.12	0.6756/5.40	0.6346/5.69	0.7835	0.5805	0.8104
User 4	0.6847/7.18	0.6665/7.74	0.7627/7	0.8297	0.7691	0.8234
